# Harm reduction behaviors are associated with carrying naloxone among patients on methadone treatment

**DOI:** 10.1186/s12954-023-00745-6

**Published:** 2023-02-14

**Authors:** Zofia Kozak, Daniel Ciccarone, Johannes Thrul, Thomas O. Cole, Alexander L. Pappas, Aaron D. Greenblatt, Christopher Welsh, Mark Yoon, Donald Gann, E. Erin Artigiani, Eric D. Wish, Annabelle M. Belcher

**Affiliations:** 1 Division of Addiction Research and Treatment, Department of Psychiatry, University of Maryland School of Medicine, 1001 W. Pratt Street, Baltimore, MD 21223 USA; 2 Department of Family and Community Medicine, University of California, San Francisco School of Medicine, San Francisco, CA USA; 3 Department of Mental Health, Bloomberg School of Public Health, Johns Hopkins University, Baltimore, MD USA; 4 Sidney Kimmel Comprehensive Cancer Center at Johns Hopkins, Baltimore, MD USA; 5 Centre for Alcohol Policy Research, La Trobe University, Melbourne, Australia; 6 Present Address: Venice Family Clinic, Venice, CA USA; 7 Division of Medical Psychology, Department of Psychiatry & Behavioral Sciences, Johns Hopkins University School of Medicine, 600 N. Wolfe Street, Baltimore, 21287 USA; 8 Center for Substance Abuse Research, University of Maryland, College Park, MD USA

**Keywords:** Harm reduction, Naloxone, Methadone, Overdose

## Abstract

**Background:**

Despite the widespread availability of naloxone, US opioid overdose rates continue to rise. The “Cascade of Care” (CoC) is a public health approach that identifies steps in achieving specific outcomes and has been used to identify gaps in naloxone carriage among individuals with opioid use disorder (OUD). We sought to apply this framework to a treatment-seeking population with OUD that may be more inclined to engage in harm reduction behaviors.

**Methods:**

Patients were recruited from an urban methadone program to complete a survey. We assessed naloxone familiarity, availability, obtainability, training, and possession, as well as naloxone carriage rates, demographics, and harm reduction behaviors. A multivariable logistic regression examined associations between naloxone carriage and individual-level factors.

**Results:**

Participants (*n* = 97) were majority male (59%), with a mean age of 48 (SD = 12), 27% had college education or higher, 64% indicated injection drug use, and 84% reported past naloxone training. All participants endorsed familiarity with naloxone, but only 42% regularly carried naloxone. The following variables were associated with carrying naloxone: White race (aOR = 2.94, 95% CI 1.02–8.52), college education (aOR = 8.11, 95% CI 1.76–37.47), and total number of self-reported harm reduction behaviors (aOR = 1.45, 95% CI 1.00–2.11).

**Conclusion:**

We found low rates of naloxone carriage among methadone-treated patients. Methadone programs provide opportunities for naloxone interventions and should target racial/ethnic minorities and individuals with lower education. The spectrum of harm reduction behaviors should be encouraged among these populations to enhance naloxone carriage.

## Introduction

In the USA, despite increased federal funding to reduce opioid-related fatalities, there was a 71% increase in overdose deaths in the 12-month period ending in October 2021 compared to the same time period in 2016 [1]. Naloxone plays a critical role in preventing lethal overdoses by reversing respiratory depression and US federal funding for naloxone distribution has increased dramatically in recent years [2], with non-medical settings being recognized as important targets for intervention [3]. Current naloxone distribution targets include both street-based community outreach programs and methadone clinics and other opioid use disorder (OUD) treatment sites.

Although naloxone distribution is a public health priority, there is a well-documented gap between the supply of naloxone being distributed and the low rate at which it is physically carried, posing a major impediment to its utility in overdose reversal [4–6]. The “Cascade of Care” (CoC) is a public health approach that identifies gaps in achieving a desired outcome such as increased naloxone carriage. In this context, it assesses progressive steps of successful naloxone distribution, including naloxone awareness, obtainability, availability, training, and possession. Due to ambiguous definitions of “possession,” there has been movement toward distinguishing between naloxone “possession” and “carry” [5], especially since carrying naloxone is a more specific indicator of its use in overdose reversals [7]. Factors associated with increased naloxone carriage include sociodemographic characteristics (female sex and housing instability), engagement with the criminal-legal system (arrests and police encounters), and substance use status (active opioid use and recent treatment of a substance or alcohol use disorder (SUD/AUD)) [4, 6, 8]. Treatment-seeking individuals may have higher rates of naloxone carriage [4], though less is known about this population.

Patients receiving methadone for their OUD are an important target for naloxone distribution as they have a high relapse risk and frequent exposure to overdose, both personally and socially [9]. Because patients often visit their methadone clinics daily for treatment, clinics provide a useful setting for naloxone delivery. It is unknown whether previously identified naloxone delivery gaps apply in such treatment settings, and it is important to clarify whether previously identified factors associated with increased naloxone carriage are reflected in treatment-seeking populations.

The goal of the current study was to identify rates of naloxone carriage and individual factors associated with carriage in a population seeking OUD treatment at an urban methadone clinic.

## Methods

### Procedures and participants

A cross-sectional survey of a clinic-based convenience sample was conducted from February 2020 to December 2020. Patients who expressed interest in being contacted for research opportunities in their initial treatment intake packet were approached in person or contacted via telephone and were offered the opportunity to participate in the survey. They were informed that any decision related to their participation in the study would have no impact on their current treatment. Eligible patients were aged 18 or older and endorsed participation in methadone treatment. Patients received financial reimbursement of $5 for completion of the survey. Data collection began mid-February 2020 but was abruptly halted due to the COVID-19 pandemic. Mandates restricting clinical research halted in-person research activities, leading to a 6-month gap in data collection from 3/12/20 to 9/21/20. After this date, all data were collected via phone. All research activities were conducted under an exempt-confirmed determination made by the University of Maryland, Baltimore Institutional Review Board (HP-00088482).

### Measures

#### Sociodemographics and substance use behavior

Sociodemographic variables assessed included self-reported age, sex, race, and education. The survey also assessed history of injection drug use.

#### Naloxone CoC

The survey assessed pillars of the naloxone CoC [6]: naloxone familiarity (“Have you heard of naloxone?”), obtainability (“Do you know where you could go to get naloxone?”), availability (“Do you think naloxone is easy to get?”), training (“Ever received training on how to give naloxone?”), possession (“Do you currently own naloxone?”), and rates of carriage (“Do you carry naloxone with you?”).

#### Harm reduction behaviors

Self-reported engagement in the following behaviors was assessed: test shots (“Do you take a small test amount of drug before using the full amount to prevent overdosing?”), observing peers with higher tolerances using drugs before them (“Do you watch others with higher tolerance use drugs before you use to prevent overdosing?”), using fentanyl test strips (“Do you use fentanyl test strips to prevent overdosing?”), and using in the company of others (“Do you use opioids only when you’re with other people to prevent overdosing?”) [10, 11].

### Analysis

Participants conducted the survey either in person (pre-COVID group) or via telephone (post-COVID group). Chi-square analysis revealed no difference between the pre-COVID (38% carriage rate) and post-COVID (52% carriage rate) cohorts (*χ*^2^ (1) = 1.75, *p* = 0.186). Thus, all data were aggregated for analysis.

We first descriptively investigated the pillars of the naloxone CoC. We then compared participant characteristics according to naloxone carriage, which is the final step in the CoC, using t-tests for continuous variables and chi-square tests for categorical variables. These bivariate tests informed a subsequent multivariable logistic regression model to examine the association of naloxone carriage with all participant characteristics, substance use, and harm reduction behaviors. Variables that met the significance threshold in bivariate analysis (*p* < 0.05) were included in the final regression model. All analyses were performed using Stata (version 16.1).

## Results

**Fig. 1:**
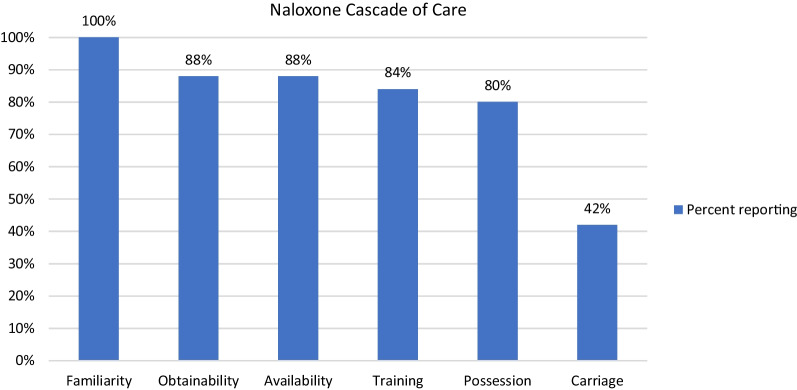
Naloxone Cascade of Care (CoC) (*N* = 97)

The sample (*n* = 97) was majority male (59%) with a mean age of 48 (SD = 12), and 65% identified as Black or African American, Asian or Pacific Islander, or “other,” 27% reported having an education of some college or greater, and 64% reported ever injecting drugs (Table 1). All participants (100%) reported familiarity with naloxone, 88% reported naloxone was readily available and easily obtainable, 84% received training on naloxone use, 80% reported possessing naloxone, and 42% reported carrying naloxone (Fig. 1).

**Table 1:** Participant characteristics for total sample and by naloxone carriage status

Variable	Overall *N* = 97	Do not carry naloxone *N* = 56	Carry naloxone *N* = 41	*p* value
Age (M, SD)	47.6 (11.9)	48.9 (12.1)	45.8 (11.6)	0.212
Sex				0.426
Female	40 (41.2%)	25 (44.6%)	15 (36.6%)	
Male	57 (58.8%)	31 (55.4%)	26 (63.4%)	
Education				**0.049**
Less than High School	19 (19.6%)	15 (26.8%)	4 (9.8%)	
High School or GED	52 (53.6%)	30 (53.6%)	22 (53.7%)	
College	26 (26.8%)	11 (19.6%)	15 (36.6%)	
Race				**0.001**
Other	63 (65.0%)	44 (78.6%)	19 (46.3%)	
White	34 (35.1%)	12 (21.4%)	22 (53.7%)	
Injected drugs				**0.040**
No	35 (36.1%)	25 (44.6%)	10 (24.4%)	
Yes	62 (63.9%)	31 (55.4%)	31 (75.6%)	
Received naloxone training				**0.037**
No	16 (16.5%)	13 (23.2%)	3 (7.3%)	
Yes	81 (83.5%)	43 (76.8%)	38 (92.7%)	
Harm reduction behaviors (M, SD)	2.5 (1.4)	2.3 (1.3)	2.9 (1.4)	**0.018**

Between-group significant differences are bolded

In bivariate analyses (Table 1), increased rates of naloxone carriage were associated with the following variables: White race (*p* = 0.001), college education (*p* = 0.018), history of ever injecting drugs (*p* = 0.043), ever receiving naloxone training (*p* = 0.048), and total number of self-reported harm reduction behaviors (*p* = 0.022).

Associations between the outcome (naloxone carriage) and all significant variables from bivariate analyses were subsequently tested in a multivariable logistic regression model (Table 2). The following variables were positively associated with carrying naloxone: White race (aOR = 2.94, 95% CI 1.02–8.52), college education (aOR = 8.11, 95% CI 1.76–37.47), and total number of self-reported harm reduction behaviors (aOR = 1.45, 95% CI 1.00–2.11).

**Table 2:** Association between patient characteristics and reported carrying of kit based on bivariate and multivariable logistic regression models (*N* = 97)

Factor	Bivariate models			Multivariable model		
	OR	95% CI	*p* value	aOR	95% CI	*p* value
Age (per year increase)	0.98	0.95, 1.01	0.211	–	–	–
Sex (female vs. male)	1.40	0.61, 3.19	0.426	–	–	–
Education (reference: less than High School)						
High School/GED	2.75	0.80, 9.43	0.108	3.71	0.93, 14.74	0.063
College	**5.11**	**1.32, 19.72**	**0.018**	**8.11**	**1.76, 37.47**	**0.007**
Race (White vs. other)	**4.25**	**1.75, 10.29**	**0.001**	**2.94**	**1.02, 8.52**	**0.046**
Injected drugs (Yes vs. No)	**2.50**	**1.03, 6.07**	**0.043**	1.68	0.57, 4.94	0.342
Ever received naloxone training (Yes vs. No)	**3.83**	**1.01, 14.47**	**0.048**	3.72	0.90, 15.49	0.071
Total harm reduction behaviors (per HR increase)	**1.46**	**1.06, 2.01**	**0.022**	**1.45**	**1.00, 2.11**	**0.049**

Significant differences are delineated in bold text

## Discussion

The current study investigated gaps in the naloxone CoC, as well as individual factors associated with likelihood of naloxone carriage in a sample of patients with OUD receiving methadone treatment. While 100% of survey participants were familiar with naloxone and its purpose, the data revealed a steep decline throughout the intermediate steps (obtainability, training, possession, and carriage), culminating in only 42% of participants reporting carrying naloxone on their person. Factors significantly associated with increased rates of naloxone carriage included White race, college education, and engaging in harm reduction behaviors.

Our results reflect previous findings wherein engagement with naloxone declines throughout the cascade [4–6, 12, 13]. In a social condition where participants interact daily with clinic staff for their treatment, and where naloxone trainings are conducted, it was surprising that less than half of the participants consistently carried naloxone. Key barriers to naloxone carriage for treatment-seeking individuals may include similar barriers experienced by non-treatment-seeking populations, including naloxone-related stigma or chaotic life circumstances that post challenges to consistent carriage. Although this number is higher than previously documented in the literature—for example, 26% naloxone carriage among a non-treatment-seeking population [6], 20% carriage rate in a systematic review [5], and 9% carriage rate in a cohort of people who use opioids (PWUO) [4]—it is surprising that we did not observe higher naloxone carriage rates in a population who accesses healthcare facilities on a regular, if not daily basis for treatment. Reasons for the higher carriage rates we observed may be explained by the fact that as treatment-seeking patients, the study participants were exposed to clinic-offered opportunities to obtain and receive training in the use of naloxone. Additionally, participants’ desire to obtain treatment may itself be linked to other protective behaviors such as naloxone carriage.

Harm reduction behaviors are important adaptive strategies used among PWUO to reduce the likelihood of lethal overdose. PWUO engage in a wide range of harm reduction behaviors such as test shots, or observing those with higher tolerances inject and using that information to gauge how much to use themselves [10, 11]. In the 2022 US National Drug Control Strategy report, the Office of National Drug Control Policy (ONDCP) acknowledges harm reduction as a critical part of the SUD delivery of care and cites naloxone distribution “especially as distributed by harm reduction organizations” as a funding priority [14]. To our knowledge, there are no published studies exploring the relationship between engaging in harm reduction behaviors and rates of naloxone carriage. Our results suggest that the probability of an individual carrying naloxone increases with each increasing harm reduction behavior they report.

We found White race was associated with increased rates of naloxone carriage, which is an extension of previous findings of racial disparities in naloxone access, including that White individuals are more likely to receive naloxone in the first place [12, 15–17]. Racial disparities in naloxone access inevitably contribute to disparities in drug overdose mortality, which have been highlighted by recent data showing American Indian or Alaska Native individuals experienced the highest rate of overdose mortality among any racial or ethnic group in 2020, and that for the first time since 1999, overdose mortality among Black individuals was higher than among White individuals [18]. Although various studies have sought to understand why PWUO are reluctant to carry naloxone even when it is free, there is a dearth of literature that focuses on barriers faced by minoritized populations. A qualitative study with a majority White PWUO population in New York City found four main barriers to naloxone possession and use: substance use stigma, indifference toward overdose, fear of negative consequences of carrying naloxone, and fear of misrecognizing an overdose and need for naloxone [19], but it is unclear whether these barriers are reflected in racial and ethnic minoritized populations. More research identifying barriers in these communities should be a public health priority, as naloxone distribution efforts will be futile without interventions tackling underlying barriers to its use.

Our finding that higher education was associated with higher rates of naloxone carriage may reflect the fact that these participants may have more psychosocial stability in terms of employment and housing which may lower the barrier to consistent carriage. Interestingly, we did not find that previous naloxone training was associated with naloxone carriage in our multivariable regression model, which further highlights the need for greater understanding of barriers to naloxone carriage, given that 84% of our sample received training. We did not find female sex to be associated with increased rates of carriage, which has been previously documented [6]. Previous research found that men who did not carry naloxone cited the kit size as a barrier to them carrying it [20], however, our findings may indicate that men have either become more accustomed to carrying naloxone, or that recent naloxone formulations changes made the packaging easier to carry. We also did not find that a history of having ever injected drugs was associated with increased rates of naloxone carriage as has been previously found [8].

This study has several limitations. These are the results of a cross-sectional survey with a convenience sample of patients with OUD participating in methadone treatment in one US city, and thus, the findings may not be generalizable. As with any survey that relies on participant self-report, our study is limited by the potential for response bias. Additionally, implementing a clinic-wide recruitment strategy allowing patients to self-elect for possible participation in research studies makes our study prone to selection bias, as we would only have enrolled individuals who were interested in engaging in studies being conducted at our site. This self-selection may have represented a distinct group of individuals who, in other ways, are not representative of the general patient population. In addition, our outcome variable (naloxone carriage) was asked using the question: “Do you carry naloxone?” The use of a single question with no qualitative descriptors (e.g., “Have you ever carried it?” vs. “Are you carrying it now?”) could be interpreted differently by individual participants [8]. Although no significant differences in naloxone carriage were found between the two groups, the fact that pre-COVID participants were interviewed in-person and those post-COVID were interviewed via telephone presents another limitation of the study as there may be differences between the two groups that were not captured. The fact that our multivariable regression model was composed of five variables significant at the bivariate level means that there may be a risk of overfitting. Finally, it is important to note that our results included large confidence intervals, specifically pertaining to race, education, and history of naloxone training. Future research with larger sample sizes should address these limitations.

In conclusion, even with consistent access to naloxone, the rate of naloxone carriage was still relatively low among treatment-seeking patients attending methadone treatment. Given their infrastructure and frequent patient contact, opioid treatment programs should be seen as crucial targets for improving rates of naloxone carriage. Finally, harm reduction behaviors should be explicitly encouraged among patients with OUD, as developing adaptive behaviors for surviving the current opioid crisis may extend into other important domains, such as carrying naloxone.

## Data Availability

The datasets used and/or analyzed during the current study are available from the corresponding author on reasonable request.

## References

[1] Ahmad FB, Rossen LM, Sutton P. Provisional drug overdose data. National Center for Health Statistics [Internet]. 2021 [cited 2022 Apr 26]. http://www.cdc.gov/nchs/nvss/vsrr/drug-overdose-data.htm.

[2] SAMHSA. SAMHSA directing $932 million to nation’s communities through the continuation of its State Opioid Response grant funding [Internet]. 2019 [cited 2022 Apr 28]. https://www.samhsa.gov/newsroom/press-announcements/201909041245.

[3] Coffin P, Sullivan S. Cost-Effectiveness of Distributing Naloxone to Heroin Users for Lay Overdose Reversal | Annals of Internal Medicine [Internet]. 2013 [cited 2022 May 16]. 10.7326/0003-4819-158-1-201301010-00003. 10.7326/0003-4819-158-1-201301010-00003 23277895

[4] Buresh M, Gicquelais RE, Astemborski J, Kirk GD, Mehta SH, Genberg BL (2020). Fatal overdose prevention and experience with naloxone: a cross-sectional study from a community-based cohort of people who inject drugs in Baltimore, Maryland. PLoS ONE.

[5] Burton G, McAuley A, Schofield J, Yeung A, Matheson C, Parkes T (2021). A systematic review and meta-analysis of the prevalence of take-home naloxone (THN) ownership and carriage. Int J Drug Policy.

[6] Tobin K, Clyde C, Davey-Rothwell M, Latkin C (2018). Awareness and access to naloxone necessary but not sufficient: examining gaps in the naloxone cascade. Int J Drug Policy.

[7] Dayton L, Gicquelais RE, Tobin K, Davey-Rothwell M, Falade-Nwulia O, Kong X (2019). More than just availability: who has access and who administers take-home naloxone in Baltimore, MD. PLoS ONE.

[8] Reed M, Wagner KD, Tran NK, Brady KA, Shinefeld J, Roth A (2019). Prevalence and correlates of carrying naloxone among a community-based sample of opioid-using people who inject drugs. Int J Drug Policy.

[9] Man LH, Best D, Gossop M, Noble A, Strang J (2002). Risk of overdose: do those who witness most overdoses also experience most overdoses?. J Subst Use.

[10] Mars SG, Ondocsin J, Ciccarone D (2018). Toots, tastes and tester shots: user accounts of drug sampling methods for gauging heroin potency. Harm Reduct J.

[11] McKnight C, Des Jarlais DC (2018). Being “hooked up” during a sharp increase in the availability of illicitly manufactured fentanyl: adaptations of drug using practices among people who use drugs (PWUD) in New York City. Int J Drug Policy.

[12] Kinnard EN, Bluthenthal RN, Kral AH, Wenger LD, Lambdin BH (2021). The naloxone delivery cascade: identifying disparities in access to naloxone among people who inject drugs in Los Angeles and San Francisco, CA. Drug Alcohol Depend.

[13] Madah-Amiri D, Gjersing L, Clausen T (2019). Naloxone distribution and possession following a large-scale naloxone programme. Addiction.

[14] The White House. National Drug Control Strategy [Internet]. Washington, D.C.: Executive Office of the President, Office of National Drug Control Policy; 2022. https://www.whitehouse.gov/wp-content/uploads/2022/04/National-Drug-Control-2022Strategy.pdf.

[15] Dayton L, Tobin K, Falade-Nwulia O, Davey-Rothwell M, Al-Tayyib A, Saleem H (2020). Racial disparities in overdose prevention among people who inject drugs. J Urban Health.

[16] Kim K, Oh H, Miller D, Veloso D, Lin J, McFarland W (2021). Prevalence and disparities in opioid overdose response training among people who inject drugs, San Francisco: naloxone training among injectors in San Francisco. Int J Drug Policy.

[17] Rowe. Predictors of participant engagement and naloxone utilization in a community‐based naloxone distribution program [Internet]. 2015 [cited 2022 Apr 26]. 10.1111/add.12961. 10.1111/add.12961 PMC4503489 25917125

[18] Friedman JR, Hansen H (2022). Evaluation of increases in drug overdose mortality rates in the US by race and ethnicity before and during the COVID-19 pandemic. JAMA Psychiatry.

[19] Bennett AS, Freeman R, Jarlais DCD, Aronson ID (2020). Reasons people who use opioids do not accept or carry no-cost naloxone: qualitative interview study. JMIR Form Res.

[20] Khatiwoda P, Proeschold-Bell RJ, Meade CS, Park LP, Proescholdbell S (2018). Facilitators and barriers to naloxone kit use among opioid-dependent patients enrolled in medication assisted therapy clinics in North Carolina. N C Med J.

